# Acute Effect of Intravenous Administration of Magnesium Sulfate on Serum Levels of Interleukin-6 and Tumor Necrosis Factor-α in Patients Undergoing Elective Coronary Bypass Graft With Cardiopulmonary Bypass

**DOI:** 10.5812/aapm.16316

**Published:** 2014-06-17

**Authors:** Parastou Aryana, Samira Rajaei, Abdolhamid Bagheri, Forouzan Karimi, Ali Dabbagh

**Affiliations:** 1 Anesthesiology Research Center, Shahid Beheshti University of Medical Sciences, Tehran, Iran; 2 Immunology Department, School of Medicine, Tehran University of Medical Sciences, Tehran, Iran; 3 Cardiology Department, School of Medicine, Shahid Beheshti University of Medical Sciences, Tehran, Iran; 4 Immunology Department, School of Medicine, Shahid Beheshti University of Medical Sciences, Tehran, Iran

**Keywords:** Magnesium, Interleukin 6, Tumor Necrosis Factor Alpha

## Abstract

**Background:**

Cardiovascular problems are among the most common health issues. A considerable number of cardiac patients undergo cardiac surgery, and coronary artery disease patients constitute about two-thirds of all these surgeries. The application of cardiopulmonary bypass (CBP) usually results in some untoward effects.

**Objectives:**

Studies have suggested magnesium sulfate (MgSO4) as an anti-inflammatory agent in a coronary artery bypass graft (CABG). This study aimed to assess the effect of an IV MgSO_4_ infusion during elective CABG (with CBP) on the blood levels of interleukin-6 (IL-6) and tumor necrosis factor alpha (TNF-α).

**Materials and Methods:**

During a 12 month period, after review board approval and based on inclusion and exclusion criteria, 90 patients were selected and entered randomly into one of the two study groups (MgSO4 or placebo). Anesthesia, surgery and CBP were performed in exactly the same way, except for the use of MgSO4 or a placebo. Both preoperative and postoperative plasma levels of IL-6 and TNF-α were checked and compared between the two groups using an ELISA.

**Results:**

There was no difference found between the two groups with regard to; gender, basic variables, Ejection Fraction (EF), CBP time and aortic cross-clamp time. The preoperative levels of IL-6 and TNF-α were not different; however, their postoperative levels were significantly higher in the placebo group (P value = 0.01 for IL-6 and 0.005 for TNF-α).

**Conclusions:**

This study showed that MgSO4 infusion could suppress part of the inflammatory response after CABG with CBP. This was demonstrated by decreased levels of interleukin-6 and TNF-α in postoperative serum levels in elective CABG with CBP.

## 1. Background

Cardiovascular problems are among the most common health problems worldwide, and they impose a significant disease burden (1-6). A considerable number of cardiac patients undergo cardiac surgery, with coronary artery disease patients constituting about two-thirds of all surgeries. This surgery usually includes the application of cardiopulmonary bypass (CBP)and it often has unwanted side effects (7-9). CBP is a common technique used every day for thousands of patients worldwide and its use has been practiced for more than 50 years (1, 2, 7, 10). Perhaps one of the most important complications of CBP is systemic inflammation caused by contact of blood with the CPB surface and this process induces widespread activation of the inflammatory pathways (1, 2, 5, 7, 11-15). Nowadays, the CBP circuits are usually coated with heparin, but the systemic inflammation resulting from the contact of blood with a foreign surface has not been resolved (1, 2, 5, 11-15). So far, several methods to control inflammation in patients undergoing bypass CBP have been proposed, those worth mentioning include; impregnation of the tubes with heparin (1, 2), administration of high doses of corticosteroids to prevent inflammation (7, 16, 17), using anti-inflammatory drugs (12), although none have proved to be totally effective (18-29).

## 2. Objectives

Recent studies have suggested that magnesium sulfate (MgSO4) solution has anti-inflammatory properties in many conditions (3-6, 30-33). In addition, it has also been demonstrated in a number of studies that magnesium can 'modulate cellular events involved in inflammation' while 'activation of leukocyte and macrophage and the release of inflammatory cytokines' are the characteristic features of this inflammatory syndrome (34, 35). Among the main proposed mechanisms for the anti-inflammatory effects of MgSO4, the 'phosphoinositide 3-kinase/Akt pathway' is one of the most important ones. Meanwhile, another main mechanism seems to be the suppressing role of magnesium throughout the inflammatory process by the 'activation of N-methyl-d-aspartate (NMDA) receptors. Since, magnesium is a natural antagonist of calcium ion and MgSO4, which acts through inhibition of 'N-methyl-D-aspartate dependent cellular pathways'(3-6, 30, 35, 36). On the other hand, it has been demonstrated that decreased plasma levels of magnesium can activate inflammatory neuromediators via the activation of 'neuroendocrinological pathways (37). At the same time, other studies have demonstrated that NF-kappaB activation with simultaneous suppression ofendotoxin, induces an increase in inflammatory mediators due to magnesium infusion in animals; furthermore, these mechanisms possibly work in a similar way in coronary artery bypass graft (CABG) patients (3-6, 34, 38-43). A number of cytokines have been named as pro-inflammatory ones; interleukin-6 (IL 6) and tumor necrosis alpha (TNF-α) are among them (26, 38, 44, 45). This randomized clinical trial study was designed and implemented to assess the effect of an intravenous magnesium sulfate (IV MgSO4) infusion compared with a placebo, during elective CABG (with CBP) on the blood levels of IL-6 and TNF-α.

## 3. Materials and Methods

The study started from October 2011 for a 12 month period, after review board approval from the Shahid Beheshti University of Medical Sciences Research Committee. The study complied with current ethical considerations. Authors declare that:

- Informed consent was obtained from each patient included in the study
- The study protocol conformed to the ethical guidelines of the 1975 Declaration of Helsinki as reflected in a priori approval by the institution's human research committee.

**Figure 1.**
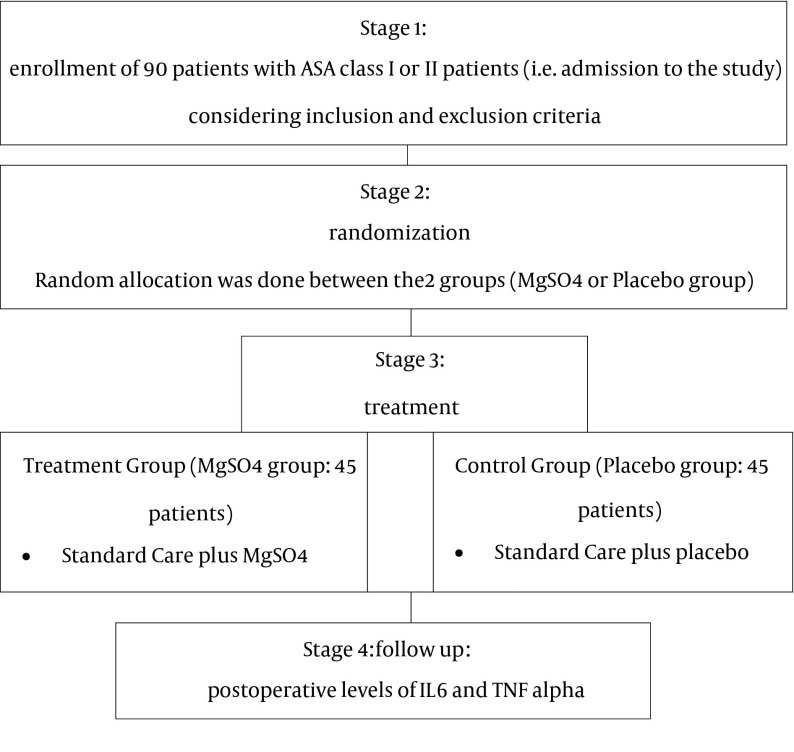
A Summary of Study Stages Are Presented in the Following Diagram

All patients in the operating room of Shahid Modarres (a university hospital affiliated to Shahid Beheshti University of Medical Sciences), undergoing elective CABG surgery were the target population, and 90 patients were selected and entered the study. The patients were randomly allocated in either the control group (45 patients) or the case group (45 patients) the case group received a MgSO4 infusion and the control group received a placebo (Figure 1). Except for this classification, there were no differences between the two groups regarding; anesthesia method, surgical procedure, surgeons and physicians, or medical treatment protocols. In addition, the volume of the magnesium infusion and the placebo as well as their syringes were similar (50 mL syringes). Sample size determination was done after a power analysis (power = 0.8, β = 0.2, α = 0.02) using sample size software: PASS 2005; NCSS, LLC; UT, USA.

Patient entry to the study was done after obtaining an informed written consent and they were allocated into the two groups based on a computer table of random numbers and after considering the inclusion and exclusion criteria.

### 3.1. Inclusion and Exclusion Criteria Were as Follows

#### 3.1.1. Inclusion Criteria

- Elective CABG using CPB
- Age 30-65 years

#### 3.1.2. Exclusion Criteria

- Underlying heart failure (right sided or left sided, including low left ventricular ejection fraction, ie. preoperative LVEF < 25%)
- Underlying renal disease
- Underlying diabetes mellitus
- Underlying uncontrolled hypertension
- Underlying thyroid problems (hypo/hyperthyroidism, active or controlled)
- Underlying malabsorption (active or controlled)
- Underlying untreated arrhythmias
- Underlying inflammatory disease (active or controlled)
- Underlying uncorrected magnesium or calcium abnormalities
- Emergent or urgent CABG
- Patient refusal to enter or continue study
- QTc interval abnormalities (including Torsades de pointes syndrome)
- Co-administration of steroids, statins, aspirin/anti-inflammatory agents

After the patient's entry into the operating room, standard monitoring was administered, including; electrocardiography (ECG), pulse oxymetry and invasive blood pressure monitoring through an indwelling arterial catheter performed after the administration of local anesthetics, and the performance of an Allen test in the non-dominant radial artery. Non-heparinized arterial samples were taken from the arterial line of the patients, and these were centrifuged and then frozen at -18°C inside the Stat laboratory of the operating room, before being collected and then transferred in an ice box at -70°C to the immunology laboratory cooperating with the study. After completion of the monitoring, the patients were anesthetized using a titrated intravenous dose of sufentanil (1 µg/Kg), cisatracurium (0.2 mg/Kg) and sodium thiopental (1 mg/Kg). Then, after 3 minutes, the patients were intubated by direct laryngoscopy. A central venous catheter was inserted through the right internal jugular approach. All the patients were anesthetized using the same method with a maintenance dose of sufentanil (0.01 µg/Kg/min), atracurium (0.5 mg/min), midazolam (1 mcg/Kg/min) and isoflurane(0.2-1%), to keep Bispectral index (BIS) levels between 40-60; the surgeon and the surgical technique were similar as far as possible. At the end of the surgery, the patients were transferred to the intensive care unit, where they were extubated after full awakening, along with full recovery of their muscle function. Furthermore, the intensive care colleague made sure that the hemostatic status of the patients was stable and did not require any surgical intervention. Then, during the 2 hour period after the surgery, non-heparinized arterial samples were taken from the arterial line of the patients, and these were centrifuged at 2500 G for 10 minutes at 4°C, then frozen at -18°C inside the Statlaboratory of the ICU, before being transferred in an ice box at -70°C to the immunology laboratory cooperating with the study. After collecting all the samples, they were thawed and used for an assessment of the serum levels of IL-1, IL-6 and TNF-α, using an ELISA assay. Each blood sampling, both before and after the operation, contained 10 ml blood without heparin. In both of the sampling steps, ie, before anesthesia and after transferring the patient to the ICU, the blood was centrifuged at 2500 G for 10 minutes at 4°C. Then, 2250 Lambda of the centrifuged plasma was separated and collected in Eppendorf microtubes. Next they were frozen at -18°C, and then transferred to -70°C, as described previously. The 2250 Lambda of the centrifuged serum was separated into 3 samples of 500 Lambda and one sample of 750. The 500 Lambda samples were used for an ELISA assessment of IL-6 and TNF-α. After completion of the sampling stage during the 6 month period, the samples were removed from the freezer, and the samples were melted in each ELISA well kit. The results of a standard ELISA kit based on optical density spectrophotometry were calculated as concentrations of interleukins. One of the members of the research team divided the patients randomly (according to the table of random numbers) into one of the two study groups: group 1 (MgSO_4_ group) or group 2 (placebo group). For the patients in group 1, a bolus dose of MgSO_4_ (30 mg/Kg in 5 minutes) was administered after the induction of anesthesia; then, the maintenance dose of MgSO_4_ (10 mg/Kg/hour) was infused. In the placebo group, the patients received normal saline with the same volume and the same infusion model. The blood samples of the patients were collected as samples of the 1st or the 2nd group and each patient was given a specific code which was noted in a data collection notebook. Meanwhile, another colleague gathered the data from the patients in the intensive care unit, in addition, she was blinded regarding the group to which the patients belonged.

The study data were recorded and extracted from each patient’s data sheet, and statistical analysis was performed using SPSS (version 11.5; SPSS Inc, Chicago, IL). For statistical data analysis, Student's t-test and Chi-square test were used. A P-value less than 0.05 was considered significant.

## 4. Results

There was no difference between the two groups regarding demographic variables including; gender, age, weight, ejection fraction (EF), cardiopulmonary bypass pump time, and aortic cross-clamp time (Table 1).

**Table 1.** Basic Variables in the Two Groups ^a^

	MgSO_4_ group	Placebo group	P value
**Gender**			> 0.05 ^b^
Male	28	30	-
Female	17	15	-
**Age, y**	64 ± 8	62 ± 10	> 0.05
**Weight, kg**	73 ± 12	77 ± 6	> 0.05
**LVEF, %**	44 ± 12	47 ± 10	> 0.05
**CPB time**	96 ± 8	92 ± 12	> 0.05
**ACC time**	54 ± 6	57 ± 7	> 0.05

^a^ Abbreviations: MgSO_4_, magnesium sulfate; CPB, cardiopulmonary bypass pump; Left Ventricle Ejection Fraction (LVEF); ACC, aortic cross-clamp.

^b^ Chi square.

In the MgSO_4_ group, the post-operative level of IL6 was 67.6 ± 22.3 pg/mL; while in the placebo group, the post-operative level of IL-6 was 102.1 ± 33.7 pg/ml (P value = 0.01). Also, the postoperative level of TNF-α in the MgSO_4_ group was 27.4 ± 4.2 pg/mL; while in the placebo group, the post-operative level of TNF-α was 44.7 ± 6.1 pg/mL (P value = 0.005).

## 5. Discussion

The results of this study demonstrated that administration of MgSO_4_ solution in adult patients undergoing elective CABG could suppress part of the inflammatory response after CABG with CPB. This was demonstrated as decreased levels of IL-6 and TNF-α in postoperative serum. However, the two groups had no difference in regard to cardiopulmonary bypass items, surgical variables, or anesthetic parameters; this indicated that the underlying clinical situations for the study were largely identical in the two groups. Magnesium may 'modulate cellular inflammation' while 'suppressing the inflammatory role of inflammatory cells and cytokines' (34, 35) through the 'activation of N-methyl-d-aspartate (NMDA) receptors', 'phosphoinositide 3-kinase/Akt pathway' and suppression of inflammatory neuromediators through the activation of 'neuro-endocrinological pathways' as the main mechanisms; since, magnesium is a natural antagonist of calcium ion (3-6, 30, 35-37, 42). The inflammatory response after CBP is one of the most significant side effects of this surgical intervention, and a number of compensatory mechanisms and approaches have been proposed, though many remain equivocal (1, 2, 10, 12, 27, 45-49). Magnesium infusion is an easy and routine therapeutic agent, and its anti-inflammatory effects are an important effect of the drug (4, 31, 35, 39, 50, 51). There are many studies denoting the therapeutic effects of magnesium infusion on many other systems, including its positive effects on the cardiovascular and respiratory system, as well as its analgesic effects (30). Since the usage of magnesium infusion could lead to improved inflammatory status, according to this study, it can be recommended that a magnesium infusion is used to suppress some of the inflammation in a cost effective way.

### 5.1. Limitations

Among the study methods, the limited assessment episodes of IL-6 and TNF-α assessments are among the main limitations of the study. Moreover, other interleukins could be helpful for making more exact assessments. The sample size was also limited.
